# Effectiveness and Safety of Botulinum Toxin Type A in Children with Musculoskeletal Conditions: What Is the Current State of Evidence?

**DOI:** 10.1155/2012/898924

**Published:** 2012-04-05

**Authors:** Noémi Dahan-Oliel, Bahar Kasaai, Kathleen Montpetit, Reggie Hamdy

**Affiliations:** ^1^Shriners Hospital for Children–Canada, Montreal, QC, Canada H3G 1A6; ^2^Division of Orthopaedic Surgery, McGill University, Montreal, QC, Canada H3G 1Y6

## Abstract

Children with musculoskeletal conditions experience muscle weakness, difficulty walking and limitations in physical activities. Standard treatment includes physiotherapy, casting, and surgery. The use of botulinum toxins appears as a promising treatment on its own, but usually as an adjunct to other treatment modalities and as an alternative to surgery. The objectives were to establish the evidence on the effectiveness, safety and functional outcome of BTX-A in children with musculoskeletal conditions. A literature search using five electronic databases identified 24 studies that met our inclusion criteria. Two randomized clinical trials were included; most studies were case studies with small sample sizes and no control group. Improvements in gait pattern, function, range of motion, reduction of co-contractions, and avoidance of surgical procedures were found following BTX-A injections. Adverse events were not reported in 10 studies, minor adverse events were reported in 13 children and there were no severe adverse events. Additional doses appear safe. BTX-A is a promising treatment adjunct in improving functional outcomes in children with musculoskeletal conditions. Future studies including larger samples, longer follow-up periods and a comparison group are required to provide evidence on the effectiveness and safety of this drug in children with musculoskeletal conditions.

## 1. Introduction

Thousands of children and adolescents across the United States suffer from musculoskeletal conditions each year [1, 2]. Common musculoskeletal conditions in children include cerebral palsy (CP), congenital muscular torticollis (CMT), Duchenne muscular dystrophy, idiopathic clubfoot, idiopathic toe walking (ITW), Legg-Calvé-Perthes disease (LCPD), limb length discrepancy, and neonatal brachial plexus palsy (NBPP). Musculoskeletal abnormalities and deformities can deprive children of physical activities, childhood experiences, and a healthy lifestyle. Besides the physical and psychosocial burden these conditions and injuries place on the child and family, these conditions also incur a financial burden for the patient and the healthcare system as multiple hospital visits are often required.

Proper and timely treatment including standard approaches such as physiotherapy, casting, bracing, and surgery is essential to ensure the child optimal growth and development. Besides these traditional modalities, the use of botulinum toxins appears as a promising treatment on its own, as an adjunct to other treatment modalities and as an alternative to surgery. Several authors have suggested the use of BTX-A in children with musculoskeletal conditions, yet the evidence supporting its safety and effectiveness is not well established. The use of BTX-A in children with CP has been widely documented [3–5] and is beyond the scope of this paper. The objectives of this systematic review were to establish the evidence on the effectiveness, safety, and functional outcome of BTX-A in children with musculoskeletal conditions.

### 1.1. Mechanisms of Action of Botulinum Toxins

Botulinum toxin is an extremely potent, naturally occurring poison resulting from the fermentation of the anaerobic spore-forming bacterium *Clostridium botulinum*. These toxins cause flaccid paralysis by blocking acetylcholine release, which is required for muscle contraction at the neuromuscular junction. Thus, the toxins have the capacity to reduce muscular activity in a dose-dependent manner. Muscle weakness occurs within a few days to one week after local injection, peaks within two weeks for several weeks, and then plateaus in milder form (the desired clinical effect) before gradually returning to baseline [6]. Clinically, this chemodenervation with muscle relaxation lasts between 12 and 16 weeks [7]. Recovery from the toxin-induced paralysis begins with resprouting of axon terminals and slow recovery of the neurons ability to release acetylcholine, which results in nerve conduction to be reestablished.

Clinical observations suggest that these neurotoxins have three mechanisms of action: paralytic, antisecretory, and analgesic (antinociceptive). A number of studies suggest that several pathways play a role in the analgesic effects of botulinum toxins, such as in conditions of pathologic muscle overactivity (dystonia and spasticity) [8] and in the role of the calcitonin gene-related peptide in the afferent signaling of pain [9]. The most effective dose is unknown although recommendations have been given [10]. The FDA has approved a total-body dose of 400 units administered every 12 to 16 weeks or at longer intervals to avoid toxicity. Identifying the appropriate muscle sites for injection is done through palpation, electrical stimulation, electromyography, ultrasound, fluoroscopy, and computerized tomography depending on the muscle size and location. A needle between 23 and 27 gauge is selected based on muscle depth, difficulty palpating the muscle, and use of electromyography. Injections may be administered under local anesthesia, conscious sedation, or general anesthesia [11].

### 1.2. Botulinum Toxin Serotypes

There are seven different serotypes of the neurotoxin, named botulinum toxin (BTX) types A to G. Although they all inhibit acetylcholine release from nerve terminals, they differ according to their intracellular protein targets, potency, dosing, and duration of effect. BTX-A is the serotype that has been most studied in terms of therapeutic application. BTX-B and BTX-F have also been used in clinical practice, but are less potent than BTX-A, and have a shorter duration of action [12]. BTX-B has also been shown to have regional and systemic anticholinergic adverse side effects, which limits its clinical use [4].

There are currently three types A and one type B brand of botulinum toxins available in the US market. In 2010, the US Food and Drug Administration (FDA) announced generic names for all of the versions of injectable botulinum toxins. This change in terminology is expected to differentiate between these different brands and provide each brand with its own identity thereby improving its clinical use and reducing errors and misinterpretation. Hence, OnabotulinumtoxinA (Botox by Allergan INC in the United States), AbobotulinumtoxinA (Dysport by Ipsen in France), IncobotulinumtoxinA (Xeomin by Merz Pharmaceuticals GmbH in Germany), and RimabotulinumtoxinB (Myobloc/Neurobloc by Solstice Neurosciences in the United States) are the new four generic names used in the USA. These new generic names have not yet been adopted by other regulatory agencies [13].

### 1.3. Clinical Use of Botulinum Toxin Type A

BTX-A was first approved by the FDA in 1989 for the treatment of strabismus and blepharospasm (two eye muscle disorders), making it the first botulinum toxin type A product approved in the world. In the USA, BTX-A is also approved to treat cervical dystonia and severe primary axillary hyperhidrosis in adults [13]. Recently, the FDA has also approved the use of BTX-A to treat increased muscle stiffness in the elbow, wrist, and finger muscles in adults with upper limb spasticity. In addition to its therapeutic uses, the same formulation of BTX-A was approved by the FDA in 2002 under the trade name Botox Cosmetic to improve the look of facial wrinkles in adults less than 65 years of age. BTX-A is one of the most widely researched medicines in the world.

Although the use of BTX-A has not yet been approved for use in children, it has been used in a variety of clinical conditions both for its neuromuscular and analgesic effects due to its safe, predictable, and reversible effects on motor weakness. These off-label indications include CMT, CP, idiopathic clubfoot, ITW, LCPD, lower limb lengthening and NBPP [14]. BTX-A is used in these children to decrease spasticity, manage postoperative pain, and improve quality of life. The most common use of BTX-A in children is for the treatment of spasticity in CP [15]. Studies have shown that BTX-A decreases muscle tone in children with upper and lower limbs spasticity associated with CP [4] and can help prevent the development of muscle contractures and bony deformities [16], as well as improve upper limb movement and function [15–17]. Patient selection, BTX-A dosing, dilution and administration, identification of muscle groups, and outcome measurement must be carefully considered [13, 16].

### 1.4. Adverse Events

Adverse events following botulinum toxin injection have been found to be mild, temporary, as well as dose and site related [12]. These may include local reactions, such as bruising and pain at the site of injection, excessive localized muscle weakness, and incontinence. Systemic effects are very rare [18] and may include flu-like symptoms, headaches, light-headedness, fever, chills, hypertension, diarrhea and abdominal pain, generalized weakness, dysphagia, dry mouth, and subsequent aspiration. These are far less common, are generally short-lived, and may result from the systemic spread of the toxin to adjacent muscles. A boxed warning label describing the spread of the toxin and its potentially life-threatening effects appears on the labels following the death of a child following BTX-A injections [13]. However, until today, no causal relationship confirmed the evidence relating this specific adverse event to the toxins [14]. Caution should be exerted when injecting BTX-A in children with preexisting swallowing or respiratory problems. Since all botulinum neurotoxins are proteins, immunoresistance may develop secondary to antibody formation. The incidence of antibody-mediated resistance in long-term treated patients ranges from 3 to 23%, depending on the patient sample, treatment regimen and toxin preparation [7]. A recent Australian study [19] prospectively documented the presence of adverse events in 334 children with CP in the month before and in the month after BTX-A injection. They found that the children had significant morbidities prior to the injection, adverse events were present in 23.2% of children, and no deaths occurred.

### 1.5. Congenital Muscular Torticollis

CMT is common and refers to unilateral contracture of the sternocleidomastoid muscle that restricts the infant's range of motion at the neck. Infants with CMT display head tilt toward the shortened side, which is often combined with rotation of the head to the opposite side [20, 21]. It is reported to occur in 1 infant in every 250–300 live births [21, 22]. Manual stretching is still the most common form of treatment, and about 90% of CMT resolves with stretching exercises [22]. When conservative treatment is ineffective, surgery is considered. However, as an alternative to invasive surgical intervention, BTX-A may be an option to increase the effectiveness of stretching on the side of the contracture and allow strengthening of overstretched and weakened muscles on the opposite side of the neck [12, 21, 22].

### 1.6. Duchenne Muscular Dystrophy

It is a progressive X-linked recessive disorder and is caused by a defective gene for dystrophin affecting approximately 1 out of every 3,600 male infants. Muscle tissue is replaced by adipose and connective tissue [23]. The proximal muscles of the lower extremities are affected first, with decreased range of motion, flexion contracture of the hip and knee, and extension contracture of the ankle. Symptoms usually appear before age 6 and typically include fatigue, muscle weakness, and progressive difficulty in walking. Braces may improve mobility and self-care function, and a wheelchair is often used by age 12. There is no known cure for Duchenne muscular dystrophy and by the late teens or twenties the condition is severe enough to shorten life expectancy. Treatment aims to control symptoms to maximize quality of life and maintain muscle strength and function. BTX-A may be indicated to decrease muscle contractures of the lower extremities and facilitate standing and mobility [14].

### 1.7. Idiopathic Clubfoot

In this congenital deformity, the hindfoot is in equinovarus and the midfoot and forefoot are adducted and supinated. Approximately 50% of cases of clubfoot are bilateral. The exact etiology remains unknown, although numerous factors have been implicated. Clubfoot is one of the most common birth defects, occurring in 1–3 per 1000 live births. A child with an untreated clubfoot will walk on the outer edge of the foot instead of the sole, develop painful callosities, be unable to wear shoes, and have painful feet that often limit activity. Nonsurgical modalities include serial manipulation and casting, such as Ponseti's technique [24, 25], as well as taping, physical therapy, and continuous passive motion. Surgery is indicated if satisfactory clinical and radiographic correction by nonsurgical methods is not obtained. BTX-A injection into the child's calf muscle is indicated to facilitate nonsurgical techniques, to supplement surgical release and to serve as an alternative to Achilles tendon lengthening by reducing the tone in the most contracted muscles [12, 14].

### 1.8. Idiopathic Toe Walking

This condition is present in children older than 3 years of age still walking on their toes without any neurological, orthopaedic, or psychiatric diseases. ITW has been estimated to occur in 7 to 24% of the childhood population [26]. Treatment recommendations include physical therapy, casting, bracing, surgical release, and Achilles tendon lengthening [14]. BTX-A may be useful to manage the contractures in cases in which toe walking recurs despite conservative and surgical treatment.

### 1.9. Legg-Calvé-Perthes Disease

LCPD is a degenerative disease of the hip joint affecting about 10.8 children of 100,000 children and is more common in boys [27]. It is characterized by an interruption of the blood supply of the head of the femur with loss of bone mass and joint deformity at the hip. The disease is typically found in young children, and it can lead to osteoarthritis in adults. Common symptoms include hip, knee, or groin pain, exacerbated by hip/leg movement, reduced range of motion at the hip and painful and limping gait. Physical activity such as standing, walking, running, and kneeling may cause severe irritation or inflammation of the damaged area. The goal of treatment is to prevent deformity of the femoral head [27] and avoid severe degenerative arthritis later on. BTX-A may be indicated to weaken selected muscles to restore muscle balance at the hip joint [14].

### 1.10. Lower Limb Lengthening

Children undergoing lower limb lengthening using an external fixator exhibit excellent results in most cases, yet the postoperative pain can be significant and often requires prolonged use of analgesics and even narcotics [28, 29]. Other aspects of the limb lengthening, such as the osteotomy and incision sites, gradual distraction increasing soft tissue tension, range of motion exercises, and gait training result in ongoing pain for weeks after the application. Appropriate pain management is essential for optimal quality of life and functional outcomes in children undergoing limb lengthening. BTX-A has been shown to reduce postoperative pain in children secondary to reduced muscle spasm [12] and may be indicated in children undergoing lower limb lengthening to alleviate spasm and pain during the lengthening process.

### 1.11. Neonatal Brachial Plexus Palsy

NBPP is defined as a flaccid paresis of the upper extremity secondary to unwanted muscular cocontraction or inappropriate activation of antagonist muscles due to increased forces of distraction to the neck during delivery. Associated injuries may include fractures to the clavicle and humerus, facial nerve palsy, and torticollis. Incidence varies between 0.38 and 3 per 1000 live births in industrialized countries and occurs more frequently in infants born over 4 kg, breech deliveries, maternal diabetes, and vacuum/forceps extraction [30]. While early physical therapy yields complete return to function in many infants, infants whose elbow flexion and shoulder abduction have not recovered before 6 months are indicated for surgical reconstruction of the brachial plexus [30]. A novel approach for children with NBPP injuries is the therapeutic use of BTX-A to inhibit unwanted cocontractions and activate antagonist muscles. Early treatment of these children with BTX-A injections may also result in stronger, normal muscles and may prevent the development of glenoid dysplasia [12, 31].

## 2. Materials and Methods

A literature search using the electronic databases MEDLINE, PubMed, Cochrane, Trip, and Web of Science for published articles in English from 1980 to September 2011 was conducted using botulinum toxin, congenital muscular torticollis, Duchenne muscular dystrophy, idiopathic clubfoot, idiopathic toe walking, Legg-Calvé-Perthes disease, lower limb lengthening, neonatal brachial plexus palsy, and relevant search terms. To be included, a study had to be written in English and to include children between 0 to 21 years of age with one of the above-mentioned musculoskeletal conditions. Review articles, editorials, commentaries, and conference proceedings were excluded. Research studies that were included in this review were classified as levels I to IV, based on the American Academy of Neurology (AAN) levels of evidence. Data was independently extracted by two reviewers (N. Dahan-Oliel and B. Kasaai).

## 3. Results

### 3.1. Search Results

**Figure 1:**
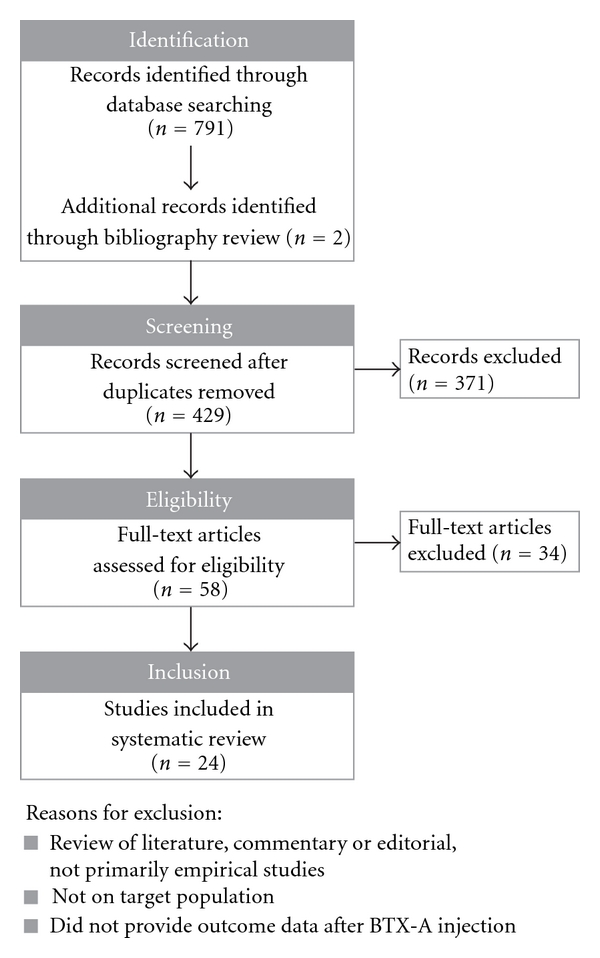


The literature search yielded 791 hits, which were then reviewed for eligibility. Twenty-four studies met our inclusion criteria and were included in this review. A flow chart illustrates the search results in Figure 1. The majority of studies were on children with NBPP (*n* = 10), followed by idiopathic clubfoot (*n* = 5), CMT (*n* = 4), ITW (*n* = 3), Duchenne muscular dystrophy (*n* = 1), and lower limb lengthening (*n* = 1). No studies that looked at the effect of BTX-A in children with LCPD were found. Findings are categorized by musculoskeletal condition and are summarized in Table 1.

### 3.2. Congenital Muscular Torticollis

Four studies were included. Two of these studies [32, 33] included both children and adults but were included as separate results for children were provided. In total, outcome was provided for forty-five children aged 3 months to 18 years following BTX-A injections. The following muscles were injected in isolation or in combination: sternocleidomastoid, trapezius, splenius, and scalenus. Head posture, cervical ROM, and pain were most frequently assessed. Improvement was reported in 36 patients (80%). Adverse events were not reported in two studies [32, 33]. Two studies reported mild dysphagia, neck weakness, neck bruising and soreness, and brief fever following injection in five children [34, 35]. There were variations in BTX-A dosage, number of injections per muscle, length of follow-up, and outcomes assessed between studies as well as within the same study, which limited comparability of findings. Standardized outcome measures were not used. It is difficult to attribute clinical improvements to the BTX-A treatment since the children in these studies often received a combination of both BTX-A injections and physical therapy. Furthermore, none of these studies compared BTX-A treatment with either traditional treatment or surgical treatment. One study on 15 infants who had a significant risk of progressing to surgery because of severe torticollis found promising results. Indeed, only the oldest infant required surgical release following BTX-A injections, while the other 14 infants showed significantly improved neck ROM and did not require surgery [35]. These findings suggest that BTX-A may be a safe and effective treatment modality when traditional treatments (home program, physical therapy) do not yield acceptable results. BTX-A treatment may obviate surgical interventions in certain cases [35], but randomized controlled trials with larger samples are needed to confirm this finding. Although it seems that the initial injections of BTX-A should be administered at a young age, the exact age is not yet established. 

### 3.3. Duchenne Muscular Dystrophy

One study met the inclusion criteria [36]. An 11-year-old boy was injected with BTX-A for tightness in the left knee flexors in order to enable standing exercises. Knee range of motion was increased by 20 degrees following injection, with no side-effects. The child's posture in the standing frame was controlled more easily. The knee range of motion decreased by 15 degrees at 5 months after injection. BTX-A may be indicated in children with Duchenne muscular dystrophy when temporary improvement of range of motion is needed to minimize knee contracture and encourage exercises for muscle stretching, prevention of osteoporosis, and retaining lung function.

### 3.4. Idiopathic Clubfoot

Five articles were included. The first study by Delgado and colleagues [41] included four patients who had severe clubfoot deformities and rapidly reached a plateau following physical therapy. BTX-A injections helped most of the patients, despite their severe deformity, allowing two of them to avoid surgery. The remaining two patients had a demyelinating neuropathy and did not respond to BTX-A treatment. Another study by Mitchell and colleagues [40] reported on three children under the age of 13 months who had a recurrence of their deformity after surgery and found a marked improvement of the deformity following BTX-A injection and application of molded plaster casts. The largest study by Alvarez and colleagues [37, 38] included 51 children. Following Ponseti-type manipulations, casting, and BTX-A injection, all but one child improved in terms of dorsiflexion and a decrease in the severity of the deformity was noted. Bracing was provided to maintain the correction. At the five-year follow-up visit, 48% (31 of 65 clubfeet) successfully responded to a single BTX-A injection and experienced no recurrence over the follow-up period. At least one repeat BTX-A injection was required in 34 clubfeet, and surgery was required in 10 clubfeet. These four studies found a positive effect of BTX-A as an adjunct to manipulation, casting, and physical therapy to correct muscle imbalance and to correct recurrent deformity in idiopathic clubfoot. All five studies concluded that BTX-A may be an effective and safe treatment alternative and can decrease the number of patients requiring surgery. A limitation of these four studies is that they did not include a control group and that the results cannot be attributed solely to BTX-A as other treatment modalities were used. Cummings and colleagues [39] conducted a randomized double-blind controlled trial on 20 infants (32 clubfeet) comparing a single BTX-A injection to placebo following serial manipulation and casting according to the Ponseti technique. The study found no significant difference in time of correction, need for tenotomy, or relapse between both groups. However, this trial included a small sample size and low BTX-A dosage that may have compromised positive findings of BTX-A. No adverse events were reported following BTX-A injections in children with clubfoot. In order to address the need for additional BTX-A injections, a larger trial is required with regular follow-up as well as a good maintenance-bracing program, so as to provide each child with an individualized treatment. The exact dose and number of injections have not been established and may vary among individuals.

### 3.5. Idiopathic Toe Walking

Three studies [42–44] reported improvement in gait pattern, function, and decreased toe walking severity following BTX-A injection to gastrocnemius and soleus muscles. Improvements were maintained at 12 months. In addition to the BTX-A injection, children also received a home exercise program, physical therapy, and orthotics. Further studies are needed to evaluate whether repeated injections and BTX treatment in combination with other treatment interventions (such as orthotics and physical therapy) improve outcome.

### 3.6. Legg-Calvé-Perthes Disease

No studies were found.

### 3.7. Lower Limb Lengthening

One study [45] met the inclusion criteria. This was a pilot randomized controlled trial comparing the effects of BTX-A injection versus placebo at the time of surgery. Fifty-two children with limb length discrepancies of various etiologies, as well as children with surgical correction of clubfoot deformities, were included. Findings showed that compared to placebo, the BTX-A group had a trend for lower pain at middistraction, less parenteral pain medication after-surgery, higher functional mobility scores, and better quality of life at three of five time points, although these differences were not statistically significant. No adverse events related to the BTX-A injection were reported, indicating that BTX-A may be safe and effective in alleviating pain, improving functional mobility and quality of life in children undergoing lower limb lengthening and/or deformity correction. Future studies with larger sample sizes are required and with homogeneous study populations to verify whether BTX-A injections are beneficial at the time of surgery in these children.

### 3.8. Neonatal Brachial Plexus Palsy

Ten studies [46–55] reported on the outcomes of using BTX-A, in which one study by Hierner and colleagues [53] was a follow-up report of the previous study by Rollnik and colleagues [55]. Although all children were diagnosed with NBPP, there were a number of variations between studies in the specific mechanism of injury and in the underlying limitations, such as in severity of biceps-triceps cocontractions, persistence in shoulder paralysis, medial rotation deformity of the shoulder, response to serial cast treatment, and posterior shoulder subluxation or dislocation. The most commonly injected muscles were pectoralis major, latissimus dorsi, and triceps. However, different muscle injection sites were used across the studies, indicating an individualized protocol according to each child's condition and needs. Four studies reported administering additional BTX-A injections, as needed. All ten studies reported positive outcomes following BTX-A injections, including improved ability to make hand-to-mouth movements, avoidance of open surgical procedures, improved active shoulder abduction and elbow extension, better functional scores, and reduction of triceps cocontractions during elbow flexion. Two studies [46, 48] reported that those children who did not experience improved outcomes following BTX-A injections were older. Desiato and Risina [54] found that the gain in shoulder abduction was directly related to younger age (*r* = 0.6). Transient weakness was reported in a 6-year-old female [54] and mild to moderate discomfort at the injection site was reported in two children [53]. Four studies reported no severe adverse event related to the BTX-A injections. Adverse events were not reported in four studies. These findings indicate that BTX-A may be a promising treatment modality in young children with NBPP; however, stronger methodologies are required to confirm the effectiveness of BTX-A for this condition.

## 4. Discussion

The objectives of this systematic review were to establish the evidence on the effectiveness of BTX-A in several musculoskeletal conditions in children and to show whether these studies reported improved functional outcome. Out of the 24 studies reviewed, only two randomized clinical trials were conducted, one in children with clubfeet [39] and the other in children undergoing lower limb lengthening [45]. Most studies were case studies with small sample sizes and no control group. Furthermore, treatment of children with musculoskeletal conditions is often multimodal, including bracing, casting, rehabilitation, and home programs. Therefore, attributing the improvements in outcome to a single intervention, such as BTX-A, is not straightforward.

 A recent systematic review on the indications for the use of BTX-A treatment for children with NBPP was conducted by Gobets and colleagues [56]. They included 10 full-text papers and six congress abstracts, involving 343 children. Four groups of indications were identified: internal rotation/adduction contracture of the shoulder, limited active elbow flexion, limited active elbow extension, and pronation contracture of the lower arm. Overall, positive results were reported for all except the indication for limited active elbow extension. However, only one study was comparative in nature; all others were classified as having a low level of evidence. There was a large variation in outcome measures. These authors conclude that multicentre randomized controlled trials are needed to provide better evidence of BTX-A in this population.

 Several factors specific to BTX-A injections may produce inter- and intrastudy variations. These factors include the different dosages and commercial sources of BTX-A used across the different studies. Two brands of BTX-A were used across the 24 studies (Allergan and Dysport). BTX-A brand was not always specified in the reviewed studies and this information was made available in several cases by contacting the authors. These two preparations should not be used interchangeably, either in terms of predicting outcome or in determining doses to be used. Though the units are not interchangeable, various published reports support a conversion ratio from 1 : 5 to 1 : 3. The technique used to identify which muscle groups will be injected also varied (palpation technique, electrical stimulation, etc.). Indeed, different studies have used different techniques and thus may lead to varying outcomes following BTX-A injection. There is no consensus as to the exact age at which a child with a musculoskeletal condition should be first administered BTX-A. Age of injection varied across the different studies reviewed. However, several authors [46, 48, 54] found that the younger children at the onset of BTX-A treatment for NBPP benefited most compared to older children. Another factor to keep in mind is the need for administering repeat BTX-A injections for sustained benefit. Some authors administered repeated BTX-A doses, whereas other authors gave just one dose, even in cases when the children did not demonstrate improved outcome following the initial BTX-A injection. This may have been due to financial and time restrictions as repeated injections are both expensive and time-consuming. Findings indicate that repeat BTX-A doses appear safe in children with musculoskeletal conditions. Length of follow-up differed across studies. Longer follow-up periods are required to inform health care providers whether the benefits of BTX-A are temporary and transient and, then if indicated, at which time should an additional injection be administered. Alvarez and colleagues [37] conducted a 5-year follow-up of their original study [38]. They reported that 48% of clubfeet were successfully corrected after a single dose of BTX-A in 44 children. This was the longest follow-up period among all the studies reviewed.

 Serious adverse events related to BTX-A injection were not reported in the studies included in this review. Minor adverse events such as transient muscular weakness and local discomfort at the injection site were reported in few studies. While this may be suggestive of the clinical safety of BTX-A, it is important to note that several studies did not actually report on the presence or absence of adverse events. Therefore, more rigor is required in reporting these events to establish the safety of BTX-A in children with musculoskeletal conditions.

## 5. Conclusions

BTX-A is a promising treatment adjunct in improving functional outcomes in children with musculoskeletal conditions by causing a flaccid paralysis of the affected muscles. Further studies should include a prospective methodology, longer follow-up periods, and comparison group and evaluate whether repeated injections are required to improve the outcome of children, thus providing evidence on the effectiveness and safety of this drug in children with musculoskeletal conditions.

##  Authors' Contributions

R. Hamdy and N. Dahan-Oliel designed the review. N. Dahan-Oliel and B. Kasaai carried out the systematic review and data extraction. K. Montpetit participated in the methodology and clinical relevance and helped draft the manuscript. All authors read and approved the final paper.

##  Conflict of Interests

The authors declare that they have no competing interests.

## Figures and Tables

**Table 1:** Findings of included studies.

Author, year	Study design	Population	Intervention and administration	Outcomes	Results	Adverse events	AAN level of evidence
Congenital Muscular Torticollis							

Bouchard et al., 2010 [32]	Case report	*N* = 3 females (age range: 15–39 y) data presented on 15 y.o. patient only	Intervention: BTX-A (Allergan). Administration: initially, 100 U in right splenius capitis + 75 U in right trapezius (for pain), then 150 U in right splenius capitis, 50 U in right trapezius, 25 U in right SCM	Muscle tightness, pain, cervical ROM Evaluated for sustained relief and sustained treatment	(i) Total resolution of abnormal head posture and pain. (ii) Regular injections during 8 years were required for sustained response and dose had to be increased.	Not reported	IV

Collins and Jankovic, 2006 [33]	Chart review	*N* = 7 children and 3 adults (age range: 6 m–41 y) data presented on 3 children (age range: 12 m–18 y)	Intervention: BTX-A (Allergan) Administration: injected into affected neck muscle (SCM, trapezius, scalenius, and splenius). Range 25–150 U administered	Pain and ROM	(i) 1 child had a moderate increase in ROM. (ii) 1 child had poor response in pain/ROM. (iii) 1 child was lost to followup.	Not reported	IV

Joyce and de Chalain, 2005 [34]	Retrospective study	*N* = 15 patients with idiopathic muscular torticollis unresponsive to traditional regimen (age range: 3–17 m (mean= 7.6 m))	Intervention: BTX-A (Allergan). Administration: injection into sternocleidomastoid and physiotherapy.Dosage: between 25 and 50 U, mean dose: 33.3 U	Subjective treatment satisfaction, ROM results	14/15 patients improved neck ROM and head position and no longer needed surgery	(i) 1 child had neck bruising. (ii) 1 child had a sore neck. (iii) 1 child had a brief fever following injection.	IV

Oleszek et al., 2005 [35]	Retrospective case series	*N* = 27 patients with CMT, (age range: 6–18 m (mean = 10.1 m))	Intervention: BTX-A (Allergan) + physical therapy for 23/25 patients Administration: injections by the same physician into sternocleidomastoid or upper trapezius muscle, or both 3 children received repeat injections. Dosage: based on age and muscle size Range 20–50 U/muscle	Qualitative comments by physician's observation Quantitative measurement by goniometer to measure changes in cervical rotation and head tilt	20/27 patients improved cervical rotation or head tilt	Two children had mild dysphagia and neck weakness	IV

Duchenne muscular dystrophy							

Von Wendt and Autti-Rämö, 1999 [36]	Case study	11 y.o. male with tightness in left knee flexors causing difficulties in standing exercises	Intervention: BTX-A (Allergan) + physical therapy twice/week + stretching of the hamstrings twice/day Administration: injection into the semitendinous muscle, semimembranous muscle, and in the biceps femoris muscle under electromyogram guidance Total dose 3 U/kg	Popliteal angle (range of motion), ability to do the standing exercises	Range of motion increased by 20 degrees after injection, but after 5 months an increase of 5 degrees compared to the initial finding was left	None	IV

Idiopathic clubfoot							

Alvarez et al., 2009 [37]	Long-term-5 year follow-up of Alvarez et al., 2005 [38]	*N* = 44 patients, with 65 idiopathic club feet (from the original 55- patient cohort)	Intervention: manipulation, casting, and BTX-A,injection (Allergan) Administration: (i) 24 patients required no additional injections from previous treatment. (ii) 29 patients required ≥2 injections.	Passive ankle ROM Dorsiflexion with 90-degree flexion (DFF), with knee in full extension (DFE), plantar flexion, and heel bisector	Over the follow-up: (i) Treated clubfeet maintained a DFF ≥ 15 degrees (ii) 48% of clubfeet were successfully corrected after single dose of BTX-A	Not reported	IV

Cummings, 2009 [39]	RCT	*N* = 32 newborns, aged 0–30 d, with 32 congenital clubfeet (Dimeglio grade III)	Intervention: the Ponseti method treatment + BTX-A (Allergan) or placebo Administration: Single injection of 7.5U BTX-A in gastrocsoleus muscle + 7.5 U in tibialis posterior muscle	Primary outcomes recorded: time required for casting and the need for Achilles tenotomy	No statistical effect found with BTX-A, as administered in this study, BTX-A did not reduce the time in cast by more than 16 days and did not reduce the need for tenotomy	Not reported	I

Alvarez et al., 2005 [38]	Prospective clinical study	*N* = 51 patients with 73 idiopathic clubfeet Group 1: initial visit < 30 days Group 2: initial visit between 30 days and 1 y	Intervention: Ponseti-type manipulations and castings, followed by BTX-A (Allergan) Administration: BTX-A (10 U/kg) injected into affected muscle Dose: total dose was divided between 2 legs for bilateral clubfeet	Pirani score and mean foot dorsiflexion scores Patients evaluated at regular intervals: ankle ROM, recurrences, and interventions for recurrences	(i) 50/51 patients had successful attenuation of triceps surae complex with major improvement in mean foot dorsiflexion score after BTX-A. (ii) Pirani scores also reduced after BTX-A by 4.45 and 3, in group 1, and 2, respectively. (iii) Mean follow-up: 12 months ±3 months.	None	III

Mitchell et al., 2004 [40]	Preliminary report	*N* = 3 patients (age range: 12-13 m) who underwent surgery but with recurrent deformity 3–6 months after index procedure	Intervention: BTX-A (Allergan) + molded plaster casts. Administration: injection into muscle groups thought to be responsible for recurrence (e.g., gastrocnemius, soleus, and tibialis)	Foot assessment via the Harrold classification	All 3 patients had marked correction in deformity following injection and casts	None	IV

Delgado et al., 2000 [41]	Case study	*N* = 4 patients with severe clubfoot (Dimeglio grade IV), who no longer responded to physical therapy	Intervention: BTX-A (Allergan). Administration: injection into the gastrocnemius and/or posterior tibial muscles (i) Dose: range 1.2 U–6.2 U/kg body weight, depending on severity and muscle type. (ii) Muscle was identified via electrical stimulation (tibia) or direct examination (gastrocnemius).	Quantitative measurements before and after treatment: Ankle ROM: degree of dorsiflexion and foot eversion	For 2/4 patients, BTX-A with physical therapy had a long-term positive effect 2/4 patients required surgical release after BTX-A and physical therapy	None	IV

Idiopathic toe walking							

Engström et al., 2010 [42]	Follow-up study	*N* = 15 patients (age range: 5–13 y) median age (9 y)	Intervention: BTX-A (Allergan) After injection, children/parents were instructed to stretch the calf 5x/week and walk on heels at least 50 steps/day Administration: bilateral injection into 4 sites in each calf under electromyogram amplifier guidance Dose: 6 U/kg, maximum of 400 U	(i) 3D gait analysis (pre, 3 weeks, 3, 6, and 12 months post). (ii) Classification of toe walking severity (pre, 12 months post). (iii) Parents rated the perceived amount of toe walking (pre, 6 and 12 month post).	(i) Significant improvement on gait analysis in 11/11 children at 12 months. (ii) 9/14 children displayed improvement on severity classification. (iii) Parents reported that 3/11 children completely ceased toe walking at 12 months.	Parents of 3 children reported moderate pain in calf muscle for 2-3 weeks after injection	III

Brunt et al., 2004 [43]	Follow-up study	*N* = 5 patients, age range 3.3–6.3 y (mean = 4.3 y).	Intervention: bilateral BTX-A (Allergan) injection, physical therapy 2x/week, and home program. Administration: gastrocnemius and soleus muscles under EMG guidance. Dose: 12 U/kg, maximum 400 U	Gait analysis (pre, mean 20 days post, and 12 months post)	Ankle EMG pattern during gait is normalized and a more normal foot-strike pattern is obtained Post injection improvement was maintained at 12 months	Not reported	III

Jacks et al., 2004 [44]	Follow-up study	*N* = 10 patients with previously failed treatment for toe walking, age range 2–17 y	Intervention: bilateral BTX-A (Allergan) injection, short leg walking casts after injection for 7 days, AFO and home stretching program, and electrical stimulation Administration: gastrocnemius and soleus muscles Dose: 10 U/kg	Pre and 1-, 3-, 6- and 12 months post: (i) Observed gait. (ii) ROM at ankle, knee, and hip. (iii) Parent report of Lower Extremity Function Assessment Test.	All 10 children had resolution of toe walking at 3 months after the initial injection One child required repeat injection with physical therapy after which toe waking resolved Improvements in overall function	None	III

Lower limb lengthening							

Hamdy et al., 2009 [45]	Multicenter RCT	*N* = 52 patients who underwent distraction osteogenesis and lower limb lengthening (age range: 5–21 y) (mean = 13.7 y)	Intervention: BTX-A (Allergan) or placebo. Administration: single injection into affected muscle group, during surgical procedure. Dose: 10 U/kg body weight, up to a maximum of 400 U of BTX-A, as recommended by FDA	Pain, medication use, quality of life, and functional mobility	Compared to placebo, BTX-A group had (i) lower Pain at middistraction. (ii) less parenteral pain medication aftersurgery. (iii) higher quality of life at 3 of the 5 time points. (iv) higher functional mobility scores. (v) These results were not statistically significant.	None related to BTX-A	I

Neonatal brachial plexus palsy							

Ezaki et al., 2010 [46]	Follow-up study	35 patients with posterior shoulder subluxation or dislocation (17 boys, 18 girls); mean age at treatment 5.7 m (3–16 m)	Intervention: BTX-A (Allergan) into the internal rotator muscles, closed reduction, and cast immobilization Administration: injections into subscapularis, teres major, and pectoralis major muscles Dose: 10 U/kg injected equally into the muscles (2-3 U/kg per muscle)	Passive external rotation of the shoulder, assessment with the Active Movement Scale or a modified Mallet scale in older children Imaging using radiography and/or ultrasonography	(i) 26/35 patients did not require early open surgical procedures to reduce the shoulders. (ii) The 11 children who experienced a redislocation even after a 2nd injection included the 3 oldest patients and the 2 infants whose parents refused further treatment.	None	III

Price et al., 2007 [47]	Retrospective review	26 patients with reconstruction for a medial rotation deformity of the shoulder 13 had BTX-A injection (mean age: 5.8 y) 13 did not have BTX-A (mean age: 4.0 y)	Intervention: BTX-A (Allergan) in the pectoralis major muscle at the end of the operation. Administration: 100 U of BTX-A in the pectoralis major muscle at the end of the operation	Modified Gilbert scale	(i) No significant difference between both groups preoperatively. (ii) Postop, those who had the BTX-A injection had significantly better Gilbert scores (*P* = 0.012) at mean follow-up of 3 years.	Not reported	IV

Basciani and Intiso, 2006 [48]	Case series	22 patients with mild brachial plexus palsy who previously underwent serial cast treatment unsuccessfully (10 males, 12 females mean age: 5.6 y)	Intervention: BTX-A (Dysport), then treated arm was fixed with plaster cast and progressively lengthened over 14 days cast was maintained for 30 days Administration: BTX-A injected into biceps brachii, brachialis, pronator teres, and pectoralis major with 22 U/kg	Muscle strength assessed using the Medical Research Council scale (grades 0–5), Mallet scales, Nine Hole Peg Test (NHPT), goniometry, at baseline, 3, 6, and 12 months	(i) Scores on NHPT significantly decreased (denoting better outcome) at 3, 6, and 12 months after injection. (ii) Mallet scores did not change, elbow extension significantly improved in all but 4 patients. (iii) These 4 children were older, and repeat injections were unsuccessful.	2 patients reported articular pain lasting 5 days after removal of the plaster cast (unrelated to the BTX-A injections)	IV

DeMatteo et al., 2006 [49]	Case series	8 patients (5 females, 3 males, mean age: 12.5 m range, 5–22 m)	Intervention: BTX-A (Allergan) in conjunction with intensive OT and a home program Administration: BTX-A injected in target muscles at total dose of 4 U/Kg/muscle into multiple sites along triceps or latissimus dorsi and pectoralis major	Active Movement Scale (AMS) and parent report of change before and after BTX-A	(i) After a single injection, all parents reported improved function. (ii) AMS scores improved significantly from pre BTX-A to 1 month (*P* = 0.014) and from before BTX-A to 4 months (*P* = 0.022).	Not reported	IV

Heise et al., 2005 [50]	Follow-up study	8 patients with clinical or electromyographic evidence of biceps-triceps cocontraction and poor elbow function (4 triceps, 4 biceps; 1 male, 7 females, age: 16 m–5 y)	Intervention: BTX-A (Allergan) + home-based physiotherapy, some did physio outside the hospital Administration: 1 injection of 2-3 U/kg of BTX-A divided in 2 or 3 sites	Muscle strength assessed using the Medical Research Council scale (grades 0–5), ability to perform hand-mouth contact in the sitting position	(i) 3/4 patients with injection to the triceps were able to perform hand-mouth contact in sitting (10 d to 6 m after injection), lasting up to 18 m. (ii) For biceps, 3/4 had improvement of elbow extension lasting 3–6 months. (iii) One child had no improvement after biceps injection and parents refused additional injection.	Not reported	III

Grossman et al., 2004 [51]	Case study	2 patients (10 m male, and 11 m female) with late nerve reconstruction for persistent shoulder paralysis following an upper brachial plexus birth injury	Intervention: BTX-A (Allergan) Administration: 10 U/kg into pectoralis major and latissimus dorsi	Modified Gilbert scale	Both scores advanced (from 1 to 4 and from 1 to 5)	Not reported	IV

Grossman et al., 2003 [52]	Follow-up study	19 patients with a combined reconstruction of the upper brachial plexus and shoulder for sequelae of birth injury, (mean age: 16 m, range: 11–29 m)	Intervention: BTX-A (Allergan) into pectoralis major (70 U) and latissimus dorsi (30 U) Administration: not specified	Modified Gilbert scale	(i) At follow-up (mean 42.7 months), all advanced by a mean of 2 grades (range: 2 to 5). (ii) Three children required reoperation, and 4 had persistent mild medial rotation contracture at follow-up.	Not reported	III

Hierner et al., 2001 [53]	Follow-up of Rollnik and colleagues' study (2000) at 18 m	6 females (range: 2–4 y)	Intervention: BTX-A (Dysport) injected into the triceps muscle at 2 sites under EMG guidance. Dose: average of 40 MU at a concentration of 25 MU/mL	Muscle force (MRC classification) and ROM, recurrence of cocontraction using EMG	At 18 m follow-up (i) mean elbow flexion was about 100° (range: 80–120°). (ii) reduction of triceps contraction during biceps activity was observed using EMG. (iii) average treatment time was 8–12 months (iv) no recurrence of cocontraction at 18 months.	Mild to moderate discomfort at the injection site for several days after injection in 2 children. No severe adverse events	III

Desiato and Risina, 2001 [54]	Prospective clinical study	50 patients with limited muscle compliance, impaired skilled movements, and dynamic deformities of the shoulder and elbow joints (26 males, 24 females, mean age: 4.8 y, range: 0.3–13.5 y)	Intervention: BTX-A (Dysport) and neurorehabilitation program using Reflex Locomotion Administration: BTX-A, 200 MU/mL injected in single site into selected muscles	ROM using goniometry, video recordings of spontaneous movements, Global Clinical Rating Scale (GCRS) These measurements were done before the first BTX-A injection, every 2 weeks for 3–9 months following injection, just before the next injection	(i) Additional injections in 30 children (ii) Active movements increased at a mean of 1.8 weeks after injection compared to baseline values (*P* < 0.05-0.01). (iii) Gain of shoulder abduction was directly related to younger age (*r* = 0.6). (iv) Videotaped recordings showed improvement in global movements. (v) Step-like increases of function using GCRS in 70% of patients. (vi) Plateau observed in remaining 30%.	6.8 y.o. a female experienced transient weakness of the adductor/internal rotator muscles after the 2nd injection, which lasted 10 days	III

Rollnik et al., 2000 [55]	Case study	6 females with severe biceps-triceps cocontractions after nerve regeneration following birth-related brachial plexus lesions (age range: 2–4 y)	BTX-A (Dysport) injected into the triceps muscle at 2 sites under EMG guidance Dose: Average of 40 MU at a concentration of 25 MU/mL	Muscle force (MRC classification) and ROM	(i) Onset of response at a mean of 8.5 days after injection (range: 4−14 days). (ii) Elbow ROM and muscle force of elbow flexion increased (*P* < 0.05). (iii) Hand-to-mouth movement improved in 5 children. (iv) EMG showed reduction of triceps cocontractions during elbow flexion. (v) No clinical recurrence of cocontractions after 1 year.	No severe adverse events	IV
